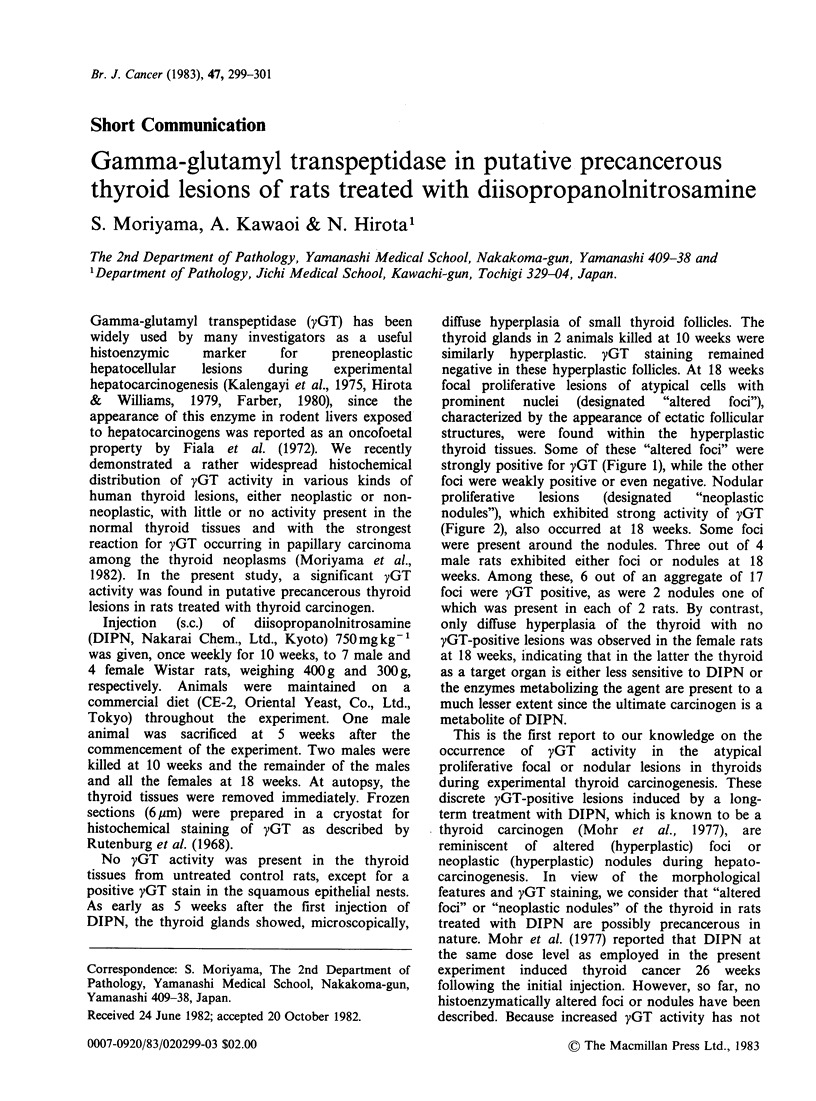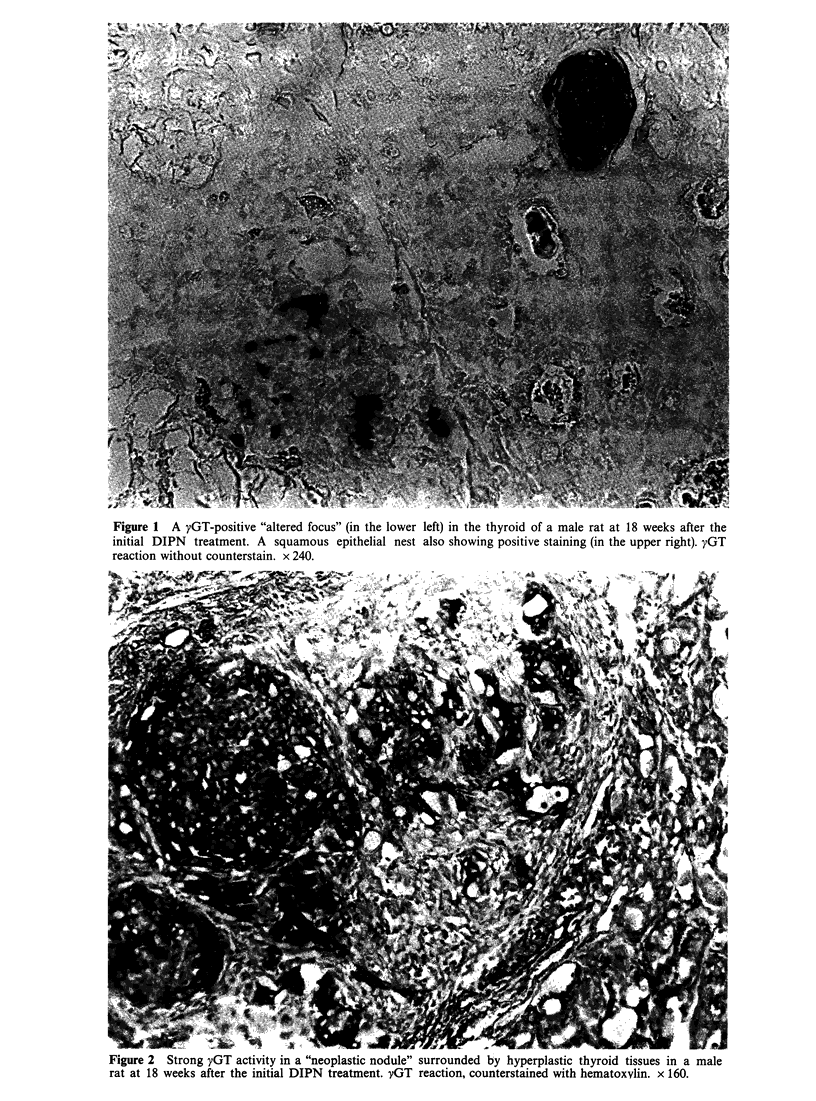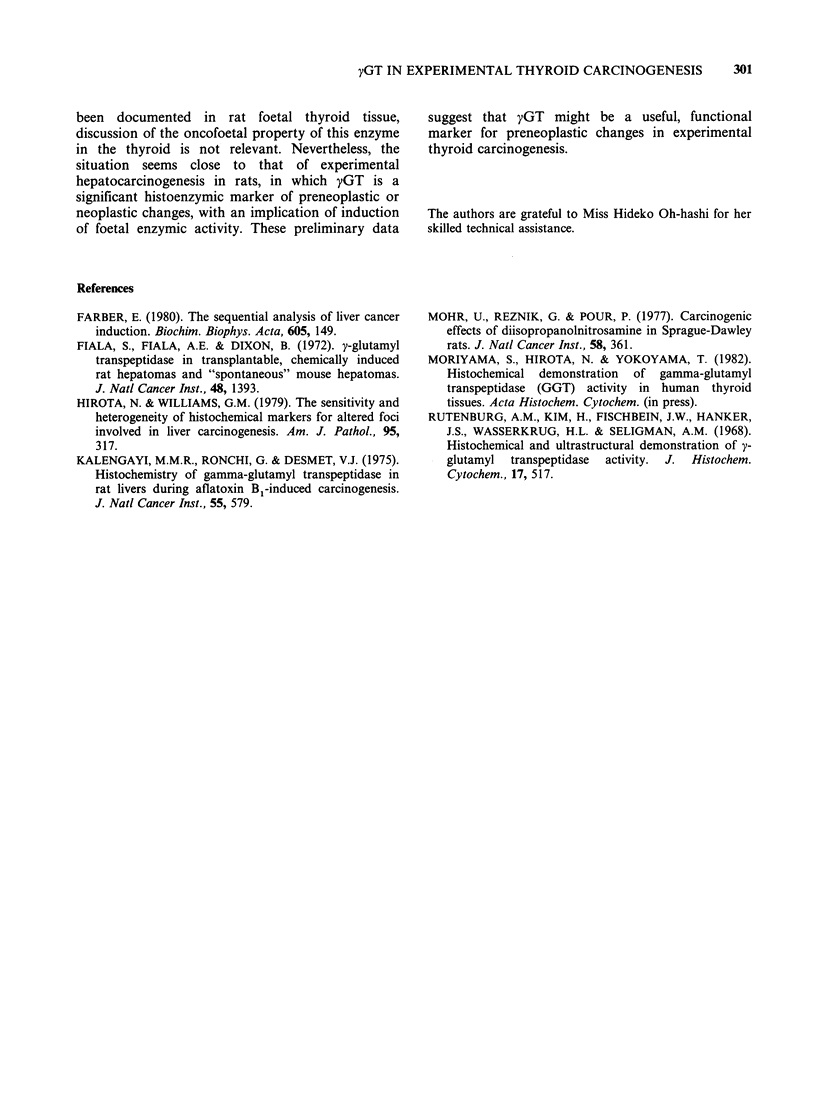# Gamma-glutamyl transpeptidase in putative precancerous thyroid lesions of rats treated with diisopropanolnitrosamine.

**DOI:** 10.1038/bjc.1983.39

**Published:** 1983-02

**Authors:** S. Moriyama, A. Kawaoi, N. Hirota

## Abstract

**Images:**


*Br. J. Cancer* (1983), **47**, 299–301

**Short Communication**

### Gamma-glutamyl transpeptidase in putative precancerous thyroid lesions of rats treated with diisopropanolnitrosamine

S. Moriyama, A. Kawaoi & N. Hirota[1]

*The 2nd Department of Pathology, Yamanashi Medical School, Nakakoma-gun, Yamanashi 409–38 and [1]Department of Pathology, Jichi Medical School, Kawachi-gun, Tochigi 329–04, Japan.*

Gamma-glutamyl transpeptidase (γGT) has been widely used by many investigators as a useful histoenzymic marker for preneoplastic hepatocellular lesions during experimental hepatocarcinogenesis (Kalengayi *et al.*, 1975, Hirota & Williams, 1979, Farber, 1980), since the appearance of this enzyme in rodent livers exposed to hepatocarcinogens was reported as an oncofoetal property by Fiala *et al.* (1972). We recently demonstrated a rather widespread histochemical distribution of γGT activity in various kinds of human thyroid lesions, either neoplastic or non-neoplastic, with little or no activity present in the normal thyroid tissues and with the strongest reaction for γGT occurring in papillary carcinoma among the thyroid neoplasms (Moriyama *et al.*, 1982). In the present study, a significant γGT activity was found in putative precancerous thyroid lesions in rats treated with thyroid carcinogen.

Injection (s.c.) of diisopropanolnitrosamine (DIPN, Nakarai Chem., Ltd., Kyoto) 750 mg kg$^{-1}$ was given, once weekly for 10 weeks, to 7 male and 4 female Wistar rats, weighing 400 g and 300 g, respectively. Animals were maintained on a commercial diet (CE-2, Oriental Yeast, Co., Ltd., Tokyo) throughout the experiment. One male animal was sacrificed at 5 weeks after the commencement of the experiment. Two males were killed at 10 weeks and the remainder of the males and all the females at 18 weeks. At autopsy, the thyroid tissues were removed immediately. Frozen sections (6 μm) were prepared in a cryostat for histochemical staining of γGT as described by Rutenburg *et al.* (1968).

No γGT activity was present in the thyroid tissues from untreated control rats, except for a positive γGT stain in the squamous epithelial nests. As early as 5 weeks after the first injection of DIPN, the thyroid glands showed, microscopically, diffuse hyperplasia of small thyroid follicles. The thyroid glands in 2 animals killed at 10 weeks were similarly hyperplastic. γGT staining remained negative in these hyperplastic follicles. At 18 weeks focal proliferative lesions of atypical cells with prominent nuclei (designated "altered foci"), characterized by the appearance of ectatic follicular structures, were found within the hyperplastic thyroid tissues. Some of these "altered foci" were strongly positive for γGT (Figure 1), while the other foci were weakly positive or even negative. Nodular proliferative lesions (designated "neoplastic nodules"), which exhibited strong activity of γGT (Figure 2), also occurred at 18 weeks. Some foci were present around the nodules. Three out of 4 male rats exhibited either foci or nodules at 18 weeks. Among these, 6 out of an aggregate of 17 foci were γGT positive, as were 2 nodules one of which was present in each of 2 rats. By contrast, only diffuse hyperplasia of the thyroid with no γGT-positive lesions was observed in the female rats at 18 weeks, indicating that in the latter the thyroid as a target organ is either less sensitive to DIPN or the enzymes metabolizing the agent are present to a much lesser extent since the ultimate carcinogen is a metabolite of DIPN.

This is the first report to our knowledge on the occurrence of γGT activity in the atypical proliferative focal or nodular lesions in thyroids during experimental thyroid carcinogenesis. These discrete γGT-positive lesions induced by a long-term treatment with DIPN, which is known to be a thyroid carcinogen (Mohr *et al.*, 1977), are reminiscent of altered (hyperplastic) foci or neoplastic (hyperplastic) nodules during hepato-carcinogenesis. In view of the morphological features and γGT staining, we consider that "altered foci" or "neoplastic nodules" of the thyroid in rats treated with DIPN are possibly precancerous in nature. Mohr *et al.* (1977) reported that DIPN at the same dose level as employed in the present experiment induced thyroid cancer 26 weeks following the initial injection. However, so far, no histoenzymatically altered foci or nodules have been described. Because increased γGT activity has not

Correspondence: S. Moriyama, The 2nd Department of Pathology, Yamanashi Medical School, Nakakoma-gun, Yamanashi 409–38, Japan.

Received 24 June 1982; accepted 20 October 1982.

0007-0920/83/020299-03 $02.00

© The Macmillan Press Ltd., 1983

**Figure 1** A $\gamma$GT-positive "altered focus" (in the lower left) in the thyroid of a male rat at 18 weeks after the initial DIPN treatment. A squamous epithelial nest also showing positive staining (in the upper right). $\gamma$GT reaction without counterstain. ×240.

**Figure 2** Strong $\gamma$GT activity in a "neoplastic nodule" surrounded by hyperplastic thyroid tissues in a male rat at 18 weeks after the initial DIPN treatment. $\gamma$GT reaction, counterstained with hematoxylin. ×160.

been documented in rat foetal thyroid tissue, discussion of the oncofoetal property of this enzyme in the thyroid is not relevant. Nevertheless, the situation seems close to that of experimental hepatocarcinogenesis in rats, in which γGT is a significant histoenzymic marker of preneoplastic or neoplastic changes, with an implication of induction of foetal enzymic activity. These preliminary data suggest that γGT might be a useful, functional marker for preneoplastic changes in experimental thyroid carcinogenesis.

The authors are grateful to Miss Hideko Oh-hashi for her skilled technical assistance.

**References**

FARBER, E. (1980). The sequential analysis of liver cancer induction. *Biochim. Biophys. Acta*, **605**, 149.

FIALA, S., FIALA, A.E. & DIXON, B. (1972). γ-glutamyl transpeptidase in transplantable, chemically induced rat hepatomas and "spontaneous" mouse hepatomas. *J. Natl Cancer Inst.*, **48**, 1393.

HIROTA, N. & WILLIAMS, G.M. (1979). The sensitivity and heterogeneity of histochemical markers for altered foci involved in liver carcinogenesis. *Am. J. Pathol.*, **95**, 317.

KALENGAYI, M.M.R., RONCHI, G. & DESMET, V.J. (1975). Histochemistry of gamma-glutamyl transpeptidase in rat livers during aflatoxin $B_1$-induced carcinogenesis. *J. Natl Cancer Inst.*, **55**, 579.

MOHR, U., REZNIK, G. & POUR, P. (1977). Carcinogenic effects of diisopropanolnitrosamine in Sprague-Dawley rats. *J. Natl Cancer Inst.*, **58**, 361.

MORIYAMA, S., HIROTA, N. & YOKOYAMA, T. (1982). Histochemical demonstration of gamma-glutamyl transpeptidase (GGT) activity in human thyroid tissues. *Acta Histochem. Cytochem.* (in press).

RUTENBURG, A.M., KIM, H., FISCHBEIN, J.W., HANKER, J.S., WASSERKRUG, H.L. & SELIGMAN, A.M. (1968). Histochemical and ultrastructural demonstration of γ-glutamyl transpeptidase activity. *J. Histochem. Cytochem.*, **17**, 517.